# Early Prediction of Seven-Day Mortality in Intensive Care Unit Using a Machine Learning Model: Results from the SPIN-UTI Project

**DOI:** 10.3390/jcm10050992

**Published:** 2021-03-02

**Authors:** Martina Barchitta, Andrea Maugeri, Giuliana Favara, Paolo Marco Riela, Giovanni Gallo, Ida Mura, Antonella Agodi, on behalf of the SPIN-UTI network

**Affiliations:** 1 Department of Medical and Surgical Sciences and Advanced Technologies “GF Ingrassia”, University of Catania, 95123 Catania, Italy; martina.barchitta@unict.it (M.B.); andrea.maugeri@unict.it (A.M.); giuliana.favara@unict.it (G.F.); 2 GISIO-SItI—Italian Study Group of Hospital Hygiene—Italian Society of Hygiene, Preventive Medicine and Public Health, 00144 Roma, Italy; idamura@uniss.it; 3 Department of Mathematics and Informatics, University of Catania, 95123 Catania, Italy; paolo.riela@unict.it (P.M.R.); gallo@dmi.unict.it (G.G.);; 4 Department of Biomedical Sciences, University of Sassari, 07100 Sassari, Italy

**Keywords:** healthcare-associated infections, machine learning, intensive care unit, risk prediction, mortality, adverse outcomes

## Abstract

Patients in intensive care units (ICUs) were at higher risk of worsen prognosis and mortality. Here, we aimed to evaluate the ability of the Simplified Acute Physiology Score (SAPS II) to predict the risk of 7-day mortality, and to test a machine learning algorithm which combines the SAPS II with additional patients’ characteristics at ICU admission. We used data from the “Italian Nosocomial Infections Surveillance in Intensive Care Units” network. Support Vector Machines (SVM) algorithm was used to classify 3782 patients according to sex, patient’s origin, type of ICU admission, non-surgical treatment for acute coronary disease, surgical intervention, SAPS II, presence of invasive devices, trauma, impaired immunity, antibiotic therapy and onset of HAI. The accuracy of SAPS II for predicting patients who died from those who did not was 69.3%, with an Area Under the Curve (AUC) of 0.678. Using the SVM algorithm, instead, we achieved an accuracy of 83.5% and AUC of 0.896. Notably, SAPS II was the variable that weighted more on the model and its removal resulted in an AUC of 0.653 and an accuracy of 68.4%. Overall, these findings suggest the present SVM model as a useful tool to early predict patients at higher risk of death at ICU admission.

## 1. Introduction

Healthcare-associated infections (HAIs) are the most frequent adverse outcome occurring when patients stay in hospital wards, especially in intensive care units (ICUs) [1,2,3,4,5,6,7,8,9]. Due to their impact on patients’ morbidity and mortality, as well as on antimicrobial resistance and assistance healthcare costs, these infections represent a major concern for public health [5,10,11,12,13,14,15]. As reported by the World Health Organization (WHO), the global burden of HAIs raises up to 15% among all hospitalized patients, with a proportion that achieves more than 30% in those who stay in ICUs [16,17,18]. 

Indeed, patients admitted to ICUs generally had a worse clinical prognosis, including prolonged hospital stays, sepsis and mortality [19]. Particularly, mortality in ICUs is two time higher among infected patients than those not infected [6,8]. This in turn depends on several patients’ factor such as the use of invasive procedures, their severity, type of infection, therapy, and microorganisms’ characteristics, including clonal spread [19,20,21,22]. Nowadays, several early warning scores have been proposed as a helpful instrument to monitor patients’ clinical deterioration and disease severity during their stay in ICUs [23,24,25,26,27]. In clinical practice, the Simplified Acute Physiology Score (SAPS) II represents the most commonly used score. Specifically, it is able to predict patients’ prognosis and to estimate their risk of HAIs, sepsis and dying, according to 17 physiological variables at ICU admission [28,29,30,31,32,33,34,35,36,37]. 

With this in mind, the complexity of HAI burden suggested the need of novel approaches aimed at early identifying patients at higher risk of adverse events in ICU [38]. Indeed, the prediction of patients at higher risk of mortality in ICU play a key role in improving patients’ survival and in implementing their management [39]. Although several traditional statistical approaches are widely used in clinical practice, modern machine learning models showed more accurate results in the early identification of patients who are more likely to die during their stay in ICU, considering different sets of risk factors [9,39,40,41,42]. For instance, support vector machine (SVM) algorithm is a supervised machine learning method with a high performance in discriminating two different classes of events, especially in many medical fields where a large amount of patient’ variables are collected [43,44]. 

In the present study, we aimed to identify and predict patients at higher risk of dying, considering their clinical and pathological characteristics at ICU admission. The primary purpose of this study is to evaluate the ability of the SAPS II to predict the risk of death after 7 days from their admission to ICU. The secondary purpose is to develop and test a machine learning algorithm, which combines the SAPS II with additional patients’ characteristics, to further improve the predicting performance. Here, we used data collected during the seven editions of the “Italian Nosocomial Infections Surveillance in Intensive Care Units” (SPIN-UTI) network, an ongoing surveillance system established by the Italian Study Group of Hospital Hygiene (GISIO) of the Italian Society of Hygiene, Preventive Medicine and Public Health (SItI) [45,46,47,48,49,50,51]. From 2006 to date, the SPIN-UTI network has collected data of nearly 20,000 patients, 4300 infections and 5300 microorganisms, which helped to feed the European surveillance network with data of HAIs in Italian ICUs [45,46,47,48,49,50,51].

## 2. Materials and Methods

### 2.1. The SPIN-UTI Project

Here, we used data collected during the seven editions of the SPIN-UTI project, which were conducted from 2016 to 2019 in Italian ICUs, using the ECDC protocol [52]. The protocols used and characteristics of 20,060 patients surveyed were fully described elsewhere [45,46,47,48,49,50,51]. In general, the SPIN-UTI project prospectively surveys patients staying in ICU for more than two days and collects data at hospital, ICU and patient level. By contrast, patients who stay in ICU less than two days are excluded a priori. The reason for their exclusion is because the primary outcome of the SPIN-UTI project is the incidence of HAIs, which by definition develop after two days of ICU stay. The study was approved by the Ethics Committee “Catania 1”, Catania, Italy (protocol numbers 111/2018/PO and 295/2019/EMPO). 

### 2.2. Definition of SAPS II and Other Predictors

In the present work, SAPS II at ICU admission was initially used as the main predictor. As previously described [35], the computation of SAPS II included the following components: Age; heart rate; systolic blood pressure; temperature; Glasgow Coma Scale; continuous positive airway pressure; PaO2; FiO2; urine output; blood urea nitrogen; sodium; potassium; bicarbonate; bilirubin; white Blood Cell; chronic diseases; type of admission. Each component was assessed within 24 h from ICU admission and the worst value was recorded. The total SAPS II was finally computed as the sum of weighted values for each component [35]. The SPIN-UTI project also collected information on patients who underwent non-surgical treatment for signs and symptoms related to the acute coronary syndrome. Moreover, we defined admission with trauma those resulting from blunt or penetrating traumatic injury to the patient, with or without surgical intervention. Instead, impaired immunity was defined as an impairment due to treatment, diseases or <500 PMN/mm3. Finally, we also collected if any antibiotic therapy was administered in the 48 h preceding ICU admission and/or during the first two days of ICU stay. The occurrence of HAI was defined according to a set of clinical and laboratory criteria that are fully described in the ECDC protocol [53].

### 2.3. Dataset of “Real” Records

We first worked only on patients with a complete assessment of the following information: Sex, patient’s origin, type of ICU admission, non-surgical treatment for acute coronary disease, surgical intervention, SAPS II, presence of invasive devices at ICU admission, trauma, impaired immunity, antibiotic therapy in 48 h before or after ICU admission and onset of HAI. The primary outcome of the current analysis was mortality within seven days from ICU admission. Accordingly, the current analysis included: (1) Patients who stayed in ICU for at least seven days and (2) those who died within two to seven days after ICU admission. By contrast, patients who stayed in ICU for less than two days, those who were discharged prior to 7 days, and those who died with the first two days were excluded. The matrix of missing values and the selection of “real” records are showed in Figures S1 and S2. Specifically, this dataset of “real” records consisted on a total of 3782 patients with complete data and meeting the inclusion criteria. The dataset described above was used for traditional statistical analyses and as test set for the machine learning algorithm. 

### 2.4. Dataset of Synthetic Records

The missing data is arguably the most common problem encountered by machine learning professionals when analyzing real-world data. The same applies for the SPIN-UTI project, where a lot of records present missing information. For instance, in the current analysis there were 61% (*n* = 12,237) of records with missing data for variables defined in the paragraph above. As many statistical models and machine learning algorithms rely on complete datasets, it is key to handle the missing data appropriately. Moreover, machine learning algorithms generally requires large datasets to be trained. For these reasons, we created a dataset of synthetic records that was used as training set for the machine learning algorithm. Accordingly, we first imputed missing data from incomplete records of the original dataset, using the K-Nearest Neighbor (K-NN) imputation method described by Malarvizhi and Thanamani [54]. Specifically, we applied the K-NN algorithm considering the Euclidean distance in the feature space for non-binary variables and the Jaccard distance for dichotomic variables. After applying two cycles of K-NN imputation to the two classes of data (i.e., alive or died patients), we recovered 3258 records. More details on methods used for data imputation are reported in the Supplementary Materials. Moreover, synthetic data were generated to balance the two classes of died and alive patients, using the Synthetic Minority Over-sampling Technique (SMOTE). In contrast to under-sampling, the oversampling approach is useful to increase the cardinality of the minority class by duplicating records. In general, the SMOTE first selects random records from the minority class population, then identifies the K-NN, and finally creates synthetic records from the random ones and the randomly selected K-NN [55]. After applying the SMOTE to 3782 “real” records, we obtained 1131 synthetic records for the class of died patients. Given that, the dataset of synthetic records, which was used as the training set, included a total of 4389 records. To confirm the goodness of the training set, we compared the distributions of primary outcome and exposure variables with those obtained from the test set (Table S1 and Figures S3–S5). 

### 2.5. Statistical Analysis

All variables of the “real” dataset were described according to their type and skewness using descriptive statistics (frequencies and percentages [%] or median and interquartile range (IQR)). In an epidemiological and descriptive point of view, we compared these variables between dead and alive patients using the Mann–Whitney U test for quantitative variables and the Chi-Squared test and Chi-Squared for trend test for qualitative variables. We first used a logistic regression model to evaluate the association of SAPS II (continuous) with death. Next, we applied a logistic regression model, also including sex (dichotomous), patient’s origin (categorical: Other ward/healthcare facility, community), type of ICU admission (categorical: Medical, surgical), non-surgical treatment for acute coronary disease (dichotomous), surgical intervention (dichotomous), presence of invasive devices at ICU admission (three dichotomous variables for urinary catheter, intubation and central venous catheter, respectively), trauma (dichotomous), impaired immunity (dichotomous), antibiotic therapy in 48 h before or after ICU admission (dichotomous). We also used Receiver Operating Characteristic (ROC) curves to assess the ability of the logistic regression models to accurately identify patients who dead from those who did not. Results were reported in terms of Area Under the Curve (AUC) and 95% Confidence Interval (95% CI). With respect to the model on SAPS II alone, we identified the best cut-off value which maximized the Youden Index. For the best cut-off value, sensitivity and specificity with their 95% CI were calculated. All tests were performed at a significance level α = 0.05 and statistical analysis was conducted using SPSS v.25 (IBM Corp., Armonk, NY, USA).

### 2.6. Machine Learning Algorithm

We next compared the predictive performance of 7-day mortality between logistic regression model and a machine learning algorithm. Specifically, the algorithm combined SAPS II with the following variables collected at ICU admission: Sex, patient’s origin, type of ICU admission, non-surgical treatment for acute coronary disease, surgical intervention, presence of intubation, presence of urinary catheter, presence of central vascular catheter; trauma, impaired immunity, and antibiotic therapy in 48 h before or after ICU admission. For the current analysis, we chosen the supervised SVM algorithm as modelling tool, which can be used for classification—especially for binary classification—and regression problems. However, our dataset was not linearly separable, not allowing to satisfy all the constraints of SVM [44]. For this reason, we used a non-linear Kernel function (i.e., the Gaussian Kernel, also called as Radial basis function Kernel, RBF). Slack variables with penalty were also introduced to satisfy all the constraints in the minimization problem of SVM [44]. More details on the machine learning algorithm are reported in the Supplementary Materials. The SVM model was trained on the training set composed by synthetic records, and then tested on the test set made of “real” records. Since patients who developed HAIs during their ICU stay are generally at higher risk of death, we also tested the SVM model on those who did not acquire HAIs within seven days from ICU admission. We also assessed the predictive performance of a SVM model, which included all variables collected at ICU admission except of SAPS II. Results are reported in terms of AUC, accuracy, sensitivity, and specificity with their 95% CI. The analyses were performed using Python and Support Vector Classification (SVC) from Sklearn 0.22.1.

## 3. Results

### 3.1. Characteristics of the Dataset of “Real” Records 

The current analysis included 3782 SPIN-UTI participants without missing data (60.2% males), surveyed from 2006 to 2019. In this subsample, the median age was 70.0 years (IQR = 20) and median SAPS II score at admission was 49 (IQR = 27). Overall, 70.9% came from other wards/hospitals and 56.9%, reported a medical type of ICU admission. In particular, 4.7% and 11.4% of patients reported trauma and/or impaired immunity, respectively. Patients who underwent antibiotic therapy, surgical intervention, or non-surgical treatment for acute coronary disease were 62.6%, 34.8%, and 9.0%, respectively. With respect to invasive devices, the presence of urinary catheter, intubation and central venous catheter was reported in 77.0%, 62.4%, and 40.5% patients, respectively. Table 1 compares characteristics of patients who died (*n* = 875; 23.1%) within seven day from ICU admission with those who were still alive (*n* = 2907; 76.9%). Specifically, patients who died were older, more likely men, and with a higher SAPS II than those who did not die. Moreover, they were also more likely to come from other ward/healthcare facility and to report a medical type of ICU admission than those alive. The first group also consisted more of patients who reported impaired immunity and less traumatic events. Instead, no differences were evident for surgical intervention, non-surgical treatment for acute coronary disease, antibiotic therapy on admission and presence of invasive devices at ICU admission. 

### 3.2. Applying Logistic Regression Models to Predict the Risk of 7-Day Mortality

**Figure 1:**
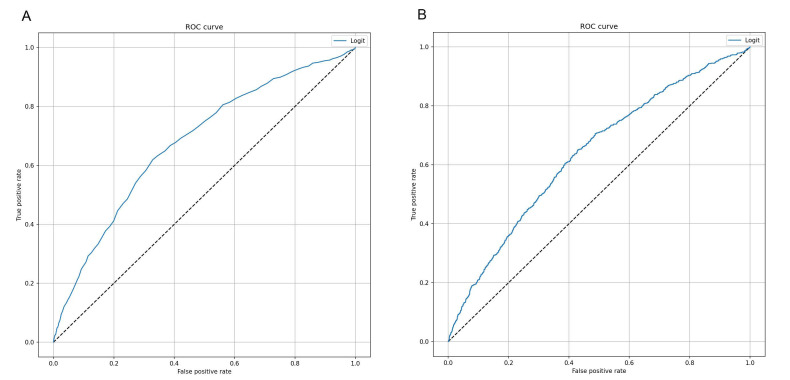
Receiver Operating Characteristic (ROC) curves of logistic regression models to predict 7-day mortality. (**A**) This curve shows the predictive performance of a logistic regression model using Simplified Acute Physiology Score (SAPS II) alone. (**B**) This curve shows the predictive performance of a logistic regression model including sex, patient’s origin, type of intensive care unit (ICU) admission, non-surgical treatment for acute coronary disease, surgical intervention, presence of invasive devices at ICU admission, trauma, impaired immunity, antibiotic therapy in 48 h before or after ICU admission.

We first applied a logistic regression model on the dataset of “real” records, using SAPS II as the independent and 7-day mortality as the dependent variable. Accordingly, Figure 1A illustrates the accuracy of SAPS II for predicting the risk of 7-day mortality for all patients admitted in ICU. We noted that SAPS II was able to discriminate patients who died from those who did not, with AUC of 0.678 (95% CI = 0.657–0.700; *p* < 0.001) and accuracy of 69.3% (95% CI = 67.8–70.8%). The coordinates of the ROC curve are reported in Table S2. Specifically, the best cut-off value of SAPS II, which maximized the Youden index, was 54.5. The application of this value resulted in sensitivity of 61.9% (95% CI = 60.4–63.4%) and specificity of 67.1% (95% CI = 65.6–68.7%). We further applied a logistic regression model, which combined SAPS II with additional patients’ characteristics collected at ICU admission. However, as indicated in Figure 1B, both AUC and accuracy of this model remained moderate (AUC = 0.637; 95% CI = 0.616–0.659; Accuracy = 65.2%; 95% CI = 63.7–66.7%). In line, sensitivity and specificity for death were 49.0% (95% CI = 47.5–50.5%), and 70.0% (95% CI = 68.5–71.5%), respectively. 

### 3.3. The SVM Algorithm Improved the Prediction of Patients Who Died

**Figure 2:**
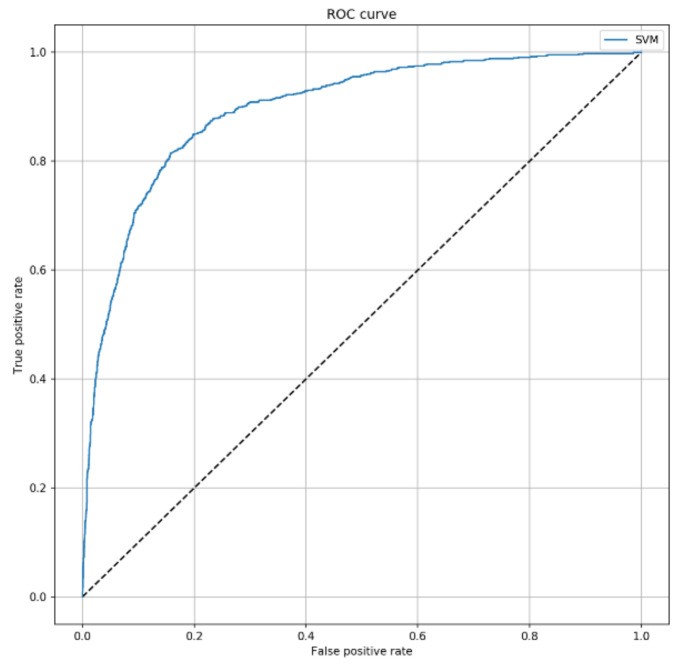
ROC curve of the Support Vector Machines (SVM) algorithm to predict 7-day mortality. This curve shows the predictive performance of the SVM algorithm including SAPS II, sex, patient’s origin, type of ICU admission, non-surgical treatment for acute coronary disease, surgical intervention, presence of invasive devices at ICU admission, trauma, impaired immunity, antibiotic therapy in 48 h before or after ICU admission. The parameters applied to the SVM algorithm were C = 2 and γ = 0.3.

Next, we aimed to develop a machine learning algorithm, which could improve the prediction of 7-day mortality in ICU. To do that, we used the SVM algorithm by combining SAPS II with other characteristics collected at ICU admission. Interestingly, the ROC curve of SVM predictive model (Figure 2) achieved an AUC of 0.896 (95% CI = 0.881–0.910; *p* < 0.001), with an accuracy of 83.5% (95% CI = 82.4–84.7%). In line, sensitivity and specificity were 81.0% (95% CI = 79.9–82.1%) and 84.0% (95% CI = 82.9–85.1%), respectively.

### 3.4. The SVM Algorithm Maintained Its Predictive Ability among Patients Who Did Not Develop HAIs

**Figure 3:**
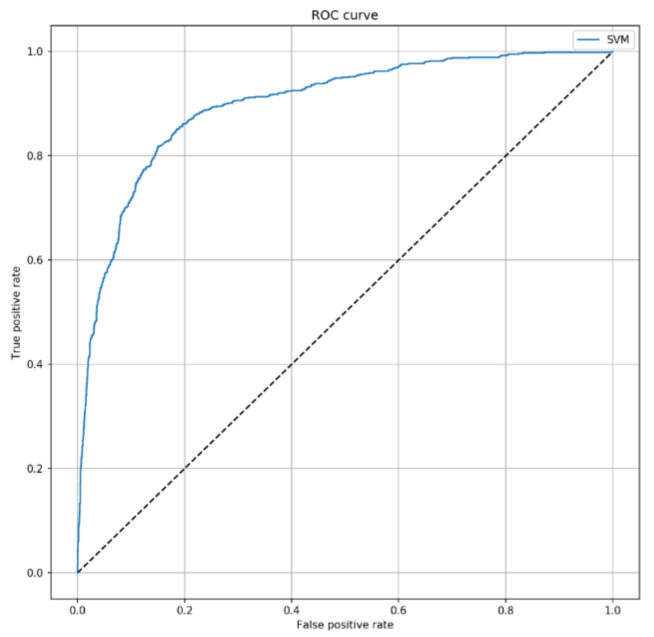
ROC curve of the SVM algorithm to predict 7-day mortality, by excluding infected patients. This curve shows the predictive performance of the SVM algorithm including SAPS II, sex, patient’s origin, type of ICU admission, non-surgical treatment for acute coronary disease, surgical intervention, presence of invasive devices at ICU admission, trauma, impaired immunity, antibiotic therapy in 48 h before or after ICU admission. The parameters applied to the SVM algorithm were C = 2 and γ=0.4.

We also tested the predictive ability of the SVM classifier among patients who did not develop HAIs within 7 days from ICU admission. To do that, we removed 520 patients with at least one HAIs from the test set. Interestingly, the model did not depend on the onset of HAI, since both AUC (0.903; 95% Confidence Interval = 0.881–0.912; *p* < 0.001) and accuracy (83.8%; 95% CI = 82.6–85.0%) remained stable (Figure 3). In line, sensitivity and specificity were comparable to those obtained in the overall analysis (82.0%; 95% CI = 80.8–83.2%; and 84.0%; 95% CI = 82.8–85.2%, respectively). 

### 3.5. The Predictivie Performance of the SVM Model by Removing SAPS II

**Figure 4:**
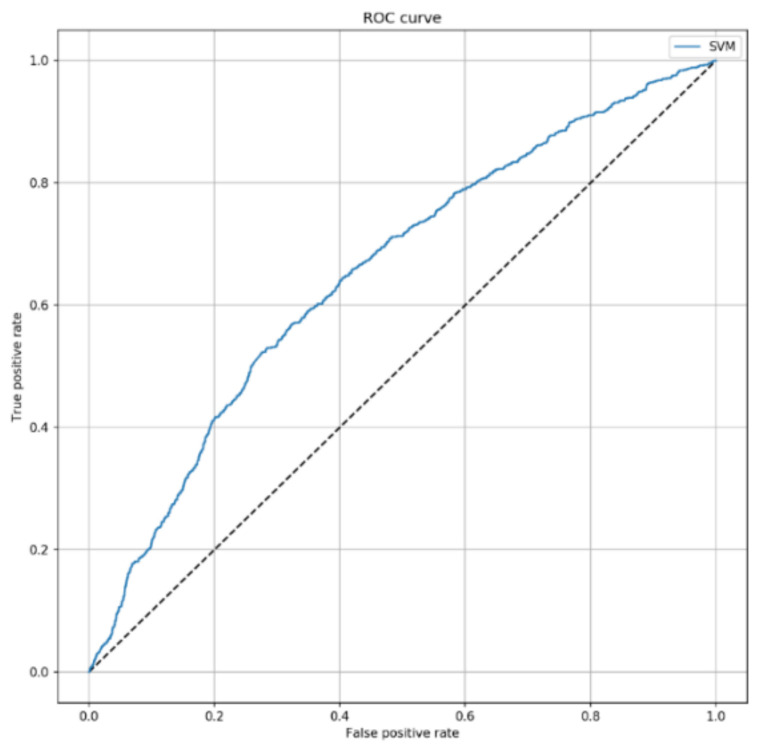
ROC curve of SVM algorithm predicting 7-day mortality, by excluding SAPS II score. This curve shows the predictive performance of the SVM algorithm including sex, patient’s origin, type of ICU admission, non-surgical treatment for acute coronary disease, surgical intervention, presence of invasive devices at ICU admission, trauma, impaired immunity, antibiotic therapy in 48 h before or after ICU admission. The parameters applied to the SVM algorithm were C = 2 and γ = 0.1.

The Shapley plot reported in Figure S6 shows the contribution of each predictors to the SVM model output. Since SAPS II was the predictor with the highest importance, we finally evaluated the predictive performance of the classifier after removing SAPS II. Interestingly, the SVM model without SAPS II led to an AUC of 0.653 (95% CI = 0.632–0.675; *p* < 0.001), with an accuracy of 68.4% (95% CI = 66.9–69.8%) on the test set (Figure 4). Accordingly, sensitivity and specificity decreased to 32.0% (95% CI = 30.5–33.5%) and 74.0% (95% CI = 72.5–75.5%), respectively. 

## 4. Discussion

In past years, numerous early warning scores were developed and employed to monitor and predict patients’ conditions and severity, as well as their adverse events in healthcare facilities [23,24,25,26,27]. Among these, SAPS II still represents one of the most widely used tool to estimate patients’ risk of death and other adverse outcomes [28,29,30,31,32,33,34,35,36,37]. In view of these considerations, we first aimed to evaluate the accuracy of SAPS II alone to identify patients who died within seven days from their admission in ICUs, using a large dataset from the SPIN-UTI project. Although AUC obtained was statistically significant, the low accuracy of nearly 69% discouraged the routinely application of SAPS II to achieve this purpose. 

Applying novel predictive algorithms, however, could be important to ameliorate patient’s safety and management in clinical practice, especially in the ICU setting. Thus, we hypothesized that combining SAPS II with other variables collected at ICU admission could improve the prediction of 7-day mortality [56,57,58,59,60]. Indeed, it is now well-established that machine learning algorithms could overcome the limitations of traditional existing tools, also allowing early prediction of mortality [9,23,24,25,26,27,40,41,42,56,61]. To do that, we developed a SVM model, which combined SAPS II with the following patients’ characteristics at ICU admission: Sex, patient’s origin, type of ICU admission, non-surgical treatment for acute coronary disease, surgical intervention, presence of invasive devices at ICU admission, trauma, impaired immunity, antibiotic therapy in 48 h before or after ICU admission and presence of infection in seven days of ICU stay. The model exhibited an AUC of 0.90 with an accuracy of 83.5% on the test set. Interestingly, its predictive performance was higher than SAPS II alone and even than a logistic regression model including additional patients’ characteristics collected at ICU admission. We also demonstrated that the performance for predicting 7-day mortality was also similar in only patients who did not acquired HAIs during their hospitalization. However, other early warning scores—for example the Acute Physiology and Chronic Health Evaluation II (APACHE II) and the Mortality Probability Model (MPM)—can assess disease severity at ICU admission and predict the risk of death, with results that were similar to SAPS II [32,62]. Thus, it will be interesting to validate our model in countries where other early warning scores are most commonly used. 

Overall, our findings underlined the potentially crucial role of machine learning algorithms in many public health issues, providing clinicians with better diagnostic tools and improving medical care in the next future. The promising benefits of applying machine learning on healthcare quality rely on the opportunity of making prevention and diagnosis as early as possible, in a context of precision medicine applicable to all settings. We recognize that findings from machine learning are frequently considered as a sort of “black box” for clinicians [63,64]. However, in our study, we aimed to use only predictors that might be related to severity and mortality of ICU patients on the basis of previous experience and expert opinion. This has certainly resulted in a more interpretable algorithm for clinicians and public health professionals [63,64]. Indeed, applying supervised machine learning algorithms could support medical professionals in parameter optimization, clustering and classification problems [21,64,65,66,67,68,69,70]. Accordingly, these algorithms—if properly applied—could overcome limitations of existing traditional early warning scores [9,23,24,25,26,27,40,41,42,56,61]. For instance, a previous study developed a machine learning algorithm based on vital signs of ICU patients, such as heart and respiration rate, oxygen saturation and blood pressure. In particular, the algorithm was able to predict mortality in ICU with an accuracy of 91.6% [71]. Our findings—together with those from other research groups—lay the foundations to develop automated and real-time tools able to identify patients who need more attention because of their high risk of death.

Our model had several strengths, including the better ability of predicting 7-day mortality if compared with an early warning score as SAPS II. Moreover, our model was trained and tested on large datasets obtained through patient-based surveillance, structured and standardized according to ECDC protocol. This should allow validation and comparison with other European countries. On the other hand, however, we cannot completely exclude historical bias due to a 14-year period of data collection. Beyond that, there were other considerations to keep in mind when interpreting our results. The first one was that our findings confirmed the importance of developing and validating early warning scores to predict the risk of death and other adverse outcomes in ICUs and other wards. Indeed, although we used several variables collected at ICU admission, the removal of SAPS II from the model significantly reduced the predictive performance. The second consideration was that machine learning requires a lot of variables and records, which are not always available in each healthcare settings. Although we used variables that can be easily collectible at ICU admission (e.g., patients’ demographic, origin, and type of admission, medical history, and disease severity), a lot of patients had both structural missing and missing at random data. While the first type of missing data could be easily managed by improving their collection in the next SPIN-UTI editions (e.g., making them mandatory), those that miss at random will continue to exist. This still remains a common issue encountered when analyzing real-world data. For this reason, we cannot completely exclude potential bias related to the high proportion of missing data. To partially manage missing data, we adopted a dual approach to generate synthetic records from those incomplete. Indeed, we created a dataset of synthetic records that was used as the training set for our machine learning algorithm. However, while it remains preferable using real data to train the algorithms, the comparison between training and test sets showed no significant differences. Finally, we recognize that our model was based on non-temporal variables related to patients’ characteristics as predictors and 7-day mortality as a sort of surrogate outcome for identifying patients who needed more attention in ICU. Thus, it will be our task to consider clinical outcomes prior to death and a time-series approach (e.g., survival analysis) for improving our model. 

## 5. Conclusions

With these considerations in mind, to the best of our knowledge, our study is the first employing the SVM algorithm to discriminate patients who died within seven days from their ICU admission from those who did not. The model showed good predictive performance, even though improvable. For this reason, further studies should be encouraged to develop and validate risk prediction models, which could help to predict adverse outcome as early as possible, and to improve patient care globally. 

## Figures and Tables

**Table 1:** Characteristics of patients with complete data according to their outcome status.

Characteristics	Patients (*n* = 3782)	Died Patients (*n* = 875)	Alive Patients (*n* = 2907)	*p*-Value
Age, years	70.0 (20.0)	74.0 (17.0)	69.0 (21.0)	<0.001
Sex (% men)	60.2%	55.0%	61.7%	<0.001
Patient’s origin				
Other ward/healthcare facility	70.9%	70.1%	71.1%	<0.001
Community	29.1%	29.9%	28.9%	
SAPS II at admission	49.0 (27.0)	59.0 (27.0)	46.0 (25.0)	<0.001
Type of ICU admission				
Medical	56.9%	59.3%	56.1%	<0.001
Surgical	43.1%	40.7%	43.9%	
Trauma	4.7%	2.4%	5.4%	<0.001
Impaired immunity	11.4%	15.0%	10.3%	<0.001
Non-surgical treatment for acute coronary disease	9.0%	10.2%	8.7%	0.174
Surgical intervention	34.8%	32.5%	35.5%	0.306
Antibiotic therapy in 48 h before or after ICU admission	62.6%	62.2%	62.8%	0.744
Presence of urinary catheter at ICU admission	77.0%	75.9%	77.4%	0.351
Presence of intubation at ICU admission	62.4%	61.7%	62.6%	0.646
Presence of central venous catheter at ICU admission	40.5%	38.5%	41.0%	0.182

* Results are reported as median (interquartile range) for continuous variables, or percentage (%) for categorical variables. Statistical analyses were performed using the Mann–Whitney, the Chi-squared test, or the Chi-squared test for trend.

## Data Availability

The data presented in this study are available on request from the corresponding author.

## Supplementary Materials

The following are available online at https://www.mdpi.com/2077-0383/10/5/992/s1, Figure S1: Matrix of missing values; Figure S2: Selection of records with complete data satisfying inclusion criteria; Figure S3: Comparison of Age (A) and SAPS II score (B) distributions between Training and Test sets, Figure S4: Comparison of dichotomous variables between Training and Test sets, Figure S5: Comparison of categorical variables between training and test datasets; Figure S6: Shapley plot showing the contribution of each predictor to the SVM model output; Table S1: Composition of training and test datasets; Table S2: Coordinates of the ROC curve of logistic regression model with SAPS II alone; Supplementary Methods for Data Imputation and Support Vector Machine model.

## References

[1] Haque M., Sartelli M., McKimm J., Bin Abu Bakar M. (2018). Health care-associated infections—An overview. Infect. Drug Resist..

[2] Revelas A. (2012). Healthcare-associated infections: A public health problem. Niger. Med. J..

[3] Hughes R.G. (2008). Patient Safety and Quality: An Evidence-Based Handbook for Nurses.

[4] Zhang Y., Du M., Johnston J.M., Andres E.B., Suo J., Yao H., Huo R., Liu Y., Fu Q. (2019). Incidence of healthcare-associated infections in a tertiary hospital in Beijing, China: Results from a real-time surveillance system. Antimicrob. Resist. Infect. Control..

[5] Allegranzi B., Nejad S.B., Combescure C., Graafmans W., Attar H., Donaldson L., Pittet D. (2011). Burden of endemic health-care-associated infection in developing countries: Systematic review and meta-analysis. Lancet.

[6] Vincent J.-L., Rello J., Marshall J.K., Silva E., Anzueto A., Martin C.D., Moreno R., Lipman J., Gomersall C., Sakr Y. (2009). International Study of the Prevalence and Outcomes of Infection in Intensive Care Units. JAMA.

[7] Alp E., Damani N. (2015). Healthcare-associated infections in Intensive Care Units: Epidemiology and infection control in low-to-middle income countries. J. Infect. Dev. Ctries..

[8] Wang W., Zhu S., He Q., Zhang R., Kang Y., Wang M., Zou K., Zong Z., Sun X. (2019). Developing a Registry of Healthcare-Associated Infections at Intensive Care Units in West China: Study Rationale and Patient Characteristics. Clin. Epidemiol..

[9] Scardoni A., Balzarini F., Signorelli C., Cabitza F., Odone A. (2020). Artificial intelligence-based tools to control healthcare associated infections: A systematic review of the literature. J. Infect. Public Health.

[10] Duval A., Obadia T., Martinet L., Boëlle P.-Y., Fleury E., Guillemot D., Opatowski L., Temime L., I-Bird study group (2018). Measuring dynamic social contacts in a rehabilitation hospital: Effect of wards, patient and staff characteristics. Sci. Rep..

[11] Lambert M.-L., Silversmit G., Savey A., Palomar M., Hiesmayr M., Agodi A., Van Rompaye B., Mertens K., Vansteelandt S. (2014). Preventable Proportion of Severe Infections Acquired in Intensive Care Units: Case-Mix Adjusted Estimations from Patient-Based Surveillance Data. Infect. Control. Hosp. Epidemiology.

[12] Barchitta M., Maugeri A., La Rosa M.C., La Mastra C., Murolo G., Agodi A. (2020). Three-Year Trends of Healthcare-Associated Infections and Antibiotic Use in Acute Care Hospitals: Findings from 2016–2018 Point Prevalence Surveys in Sicily, Italy. Antibiotics.

[13] Barchitta M., Maugeri A., La Rosa M.C., La Mastra C., Murolo G., Basile G., Agodi A. (2020). Carbapenem Consumption and Rate of carbapenem-resistant gram-negative bacteria: Results from the Sicilian Surveillance System. Ann. Ig..

[14] Barchitta M., Quattrocchi A., Maugeri A., La Rosa M.C., La Mastra C., Sessa L., Cananzi P., Murolo G., Oteri A., Basile G. (2019). Antibiotic Consumption and Resistance during a 3-Year Period in Sicily, Southern Italy. Int. J. Environ. Res. Public Health.

[15] Agodi A., Barchitta M., Quattrocchi A., Maugeri A., Aldisio E., Marchese A.E., Mattaliano A.R., Tsakris A. (2015). Antibiotic trends of Klebsiella pneumoniae and Acinetobacter baumannii resistance indicators in an intensive care unit of Southern Italy, 2008–2013. Antimicrob. Resist. Infect. Control..

[16] Sulzgruber P., Schnaubelt S., Koller L., Laufer G., Pilz A., Kazem N., Winter M.-P., Steinlechner B., Andreas M., Fleck T. (2020). An Extended Duration of the Pre-Operative Hospitalization is Associated with an Increased Risk of Healthcare-Associated Infections after Cardiac Surgery. Sci. Rep..

[17] Zimlichman E., Henderson D., Tamir O., Franz C., Song P., Yamin C.K., Keohane C., Denham C.R., Bates D.W. (2013). Health Care–Associated Infections. JAMA Intern. Med..

[18] World Health Organization (2011). Report on the Burden of Endemic Health Care-Associated Infection Worldwide.

[19] Garrouste-Orgeas M., Timsit J.F., Tafflet M., Misset B., Zahar J.-R., Soufir L., Lazard T., Jamali S., Mourvillier B., Cohen Y. (2006). Excess Risk of Death from Intensive Care Unit--Acquired Nosocomial Bloodstream Infections: A Reappraisal. Clin. Infect. Dis..

[20] Alexopoulos E.C., Batzi E., Messolora F., Jelastopulu E. (2010). Wide range of point prevalences of healthcare-associated infections in Western Greece. Epidemiol. Infect..

[21] Barchitta M., Maugeri A., Favara G., Riela P., La Mastra C., La Rosa M., Lio R.M.S., Gallo G., Mura I., Agodi A. (2021). Cluster analysis identifies patients at risk of catheter-associated urinary tract infections in intensive care units: Findings from the SPIN-UTI Network. J. Hosp. Infect..

[22] Zarrilli R., Di Popolo A., Bagattini M., Giannouli M., Martino D., Barchitta M., Quattrocchi A., Iula V., De Luca C., Scarcella A. (2012). Clonal spread and patient risk factors for acquisition of extensively drug-resistant Acinetobacter baumannii in a neonatal intensive care unit in Italy. J. Hosp. Infect..

[23] Gerry S., Bonnici T., Birks J., Kirtley S., Virdee P.S., Watkinson P.J., Collins G.S. (2020). Early warning scores for detecting deterioration in adult hospital patients: Systematic review and critical appraisal of methodology. BMJ.

[24] Brennan T.A., Leape L.L., Laird N.M., Hebert L., Localio A.R., Lawthers A.G., Newhouse J.P., Weiler P.C., Hiatt H.H. (2004). Incidence of adverse events and negligence in hospitalized patients: Results of the Harvard Medical Practice Study I. Qual. Saf. Health Care.

[25] Kohn L.T., Corrigan J.M., Donaldson M.S., Institute of Medicine (US) Committee on Quality of Health Care in America (2000). To Err is Human: Building a Safer Health System.

[26] Vincent C., Neale G., Woloshynowych M. (2001). Adverse events in British hospitals: Preliminary retrospective record review. BMJ.

[27] Hillman K.M., Bristow P.J., Chey T., Daffurn K., Jacques T., Norman S.L., Bishop G.F., Simmons G. (2002). Duration of life-threatening antecedents prior to intensive care admission. Intensiv. Care Med..

[28] Allyn J., Ferdynus C., Bohrer M., Dalban C., Valance D., Allou N. (2016). Simplified Acute Physiology Score II as Predictor of Mortality in Intensive Care Units: A Decision Curve Analysis. PLoS ONE.

[29] Gilani M.T., Razavi M., Azad A.M. (2014). A comparison of Simplified Acute Physiology Score II, Acute Physiology and Chronic Health Evaluation II and Acute Physiology and Chronic Health Evaluation III scoring system in predicting mortality and length of stay at surgical intensive care unit. Niger. Med. J..

[30] Sadaka F., EthmaneAbouElMaali C., Cytron M.A., Fowler K., Javaux V.M., O’Brien J. (2017). Predicting Mortality of Patients With Sepsis: A Comparison of APACHE II and APACHE III Scoring Systems. J. Clin. Med. Res..

[31] Mungan I., Bektaş S., Çavuş M.A., Sarı S., Turan S. (2019). The predictive power of SAPS-3 and SOFA scores and their relations with patient outcomes in the Surgical Intensive Care Unit. Turk. J. Surg..

[32] Haddadi A., Ledmani M., Gainier M., Hubert H., Tagne J., De Micheaux P. (2014). Comparing the APACHE II, SOFA, LOD, and SAPS II scores in patients who have developed a nosocomial infection. Bangladesh Crit. Care J..

[33] Agodi A., Barchitta M., Auxilia F., Brusaferro S., D’Errico M.M., Montagna M.T., Pasquarella C., Tardivo S., Arrigoni C., Fabiani L. (2018). Epidemiology of intensive care unit-acquired sepsis in Italy: Results of the SPIN-UTI network. Ann. Ig. Med. Prev. Comunita.

[34] Beck D.H., Smith G.B., Pappachan J.V., Millar B. (2003). External validation of the SAPS II, APACHE II and APACHE III prognostic models in South England: A multicentre study. Intensiv. Care Med..

[35] Le Gall J.R. (1993). A new Simplified Acute Physiology Score (SAPS II) based on a European/North American multicenter study. JAMA.

[36] Nielsen A.B., Thorsen-Meyer H.-C., Belling K., Nielsen A.P., Thomas C.E., Chmura P.J., Lademann M., Moseley P.L., Heimann M., Dybdahl L. (2019). Survival prediction in intensive-care units based on aggregation of long-term disease history and acute physiology: A retrospective study of the Danish National Patient Registry and electronic patient records. Lancet Digit. Health.

[37] Knaus W.A., Draper E.A., Wagner D.P., Zimmerman J.E. (1985). APACHE II: A severity of disease classification system. Crit. Care Med..

[38] Favara G., Riela P.M., Maugeri A., Barchitta M., Gallo G., Agodi A. Risk of Pneumonia and Associated Outcomes in Intensive Care Unit: An Integrated Approach of Visual and Cluster Analysis. Proceedings of the 2019 IEEE World Congress Services.

[39] Kong G., Lin K., Hu Y. (2020). Using machine learning methods to predict in-hospital mortality of sepsis patients in the ICU. BMC Med Inform. Decis. Mak..

[40] Parreco J.P., Hidalgo A.E., Badilla A.D., Ilyas O., Rattan R. (2018). Predicting central line-associated bloodstream infections and mortality using supervised machine learning. J. Crit. Care.

[41] Deo R.C. (2015). Machine Learning in Medicine. Circulation.

[42] Deo R.C. (2020). Machine Learning in Medicine. Circulation.

[43] Yu W., Liu T., Valdez R., Gwinn M., Khoury M.J. (2010). Application of support vector machine modeling for prediction of common diseases: The case of diabetes and pre-diabetes. BMC Med. Inform. Decis. Mak..

[44] Cortes C., Vapnik V. (1995). Support-Vector Networks. Mach. Learn..

[45] Agodi A., Barchitta M., Quattrocchi A., Spera E., Gallo G., Auxilia F., Brusaferro S., D’Errico M.M., Montagna M.T., Pasquarella C. (2017). Preventable proportion of intubation-associated pneumonia: Role of adherence to a care bundle. PLoS ONE.

[46] Agodi A., Auxilia F., Barchitta M., Brusaferro S., D’Errico M.M., Montagna M.T., Pasquarella C., Tardivo S., Mura I. (2015). Antibiotic consumption and resistance: Results of the SPIN-UTI project of the GISIO-SItI. Epidemiol. Prev..

[47] Agodi A., Auxilia F., Barchitta M., Brusaferro S., D’Alessandro D., Grillo O., Montagna M., Pasquarella C., Righi E., Tardivo S. (2013). Trends, risk factors and outcomes of healthcare-associated infections within the Italian network SPIN-UTI. J. Hosp. Infect..

[48] Agodi A., Auxilia F., Barchitta M., Brusaferro S., D’Alessandro D., Montagna M., Orsi G.B., Pasquarella C., Torregrossa V., Suetens C. (2010). Building a benchmark through active surveillance of intensive care unit-acquired infections: The Italian network SPIN-UTI. J. Hosp. Infect..

[49] Agodi A., Auxilia F., Barchitta M., D’Errico M.M., Montagna M.T., Pasquarella C., Tardivo S., Mura I. (2014). Control of intubator associated pneumonia in intensive care unit: Results of the GISIO-SItI SPIN-UTI Project. Epidemiol. Prev..

[50] Agodi A., Barchitta M., Mura I., Pasquarella C., Torregrossa M.V., SItI G. (2018). The commitment of the GISIO-SItI to contrast Healthcare-Associated Infections and the experience of prevalence studies in Sicily. Ann. Ig..

[51] Masia M., Barchitta M., Liperi G., Cantù A., Alliata E., Auxilia F., Torregrossa V., Mura I., Agodi A. (2010). Validation of intensive care unit-acquired infection surveillance in the Italian SPIN-UTI network. J. Hosp. Infect..

[52] European Center for Disease Prevention and Control (2015). European Surveillance of Healthcare-Associated Infections in Intensive Care Units- HAI-Net ICU Protocol- Protocol Version 1.02.

[53] European Centre for Disease Prevention and Control (2010). European Surveillance of Healthcare-Associated Infections in Intensive Care Units.

[54] Malarvizhi R., Thanamani A. (2012). K-nearest neighbor in missing data imputation. Int. J. Eng. Res. Dev..

[55] Chawla N.V., Bowyer K.W., Hall L.O., Kegelmeyer W.P. (2002). SMOTE: Synthetic minority over-sampling technique. J. Artif. Intell. Res..

[56] Lovejoy C.A., Buch V., Maruthappu M. (2019). Artificial intelligence in the intensive care unit. Crit. Care.

[57] Strand K., Flaatten H. (2008). Severity scoring in the ICU: A review. Acta Anaesthesiol. Scand..

[58] Komorowski M. (2019). Artificial intelligence in intensive care: Are we there yet?. Intensiv. Care Med..

[59] Fralick M., Colak E., Mamdani M. (2019). Machine Learning in Medicine. New Engl. J. Med..

[60] Meiring C., Dixit A., Harris S., Maccallum N.S., Brealey D.A., Watkinson P.J., Jones A., Ashworth S., Beale R., Brett S.J. (2018). Optimal intensive care outcome prediction over time using machine learning. PLoS ONE.

[61] Desautels T., Calvert J., Hoffman J., Jay M., Kerem Y., Shieh L., Shimabukuro D., Chettipally U., Feldman M.D., Barton C. (2016). Prediction of Sepsis in the Intensive Care Unit With Minimal Electronic Health Record Data: A Machine Learning Approach. JMIR Med. Inform..

[62] Vincent J.-L., Moreno R. (2010). Clinical review: Scoring systems in the critically ill. Crit. Care.

[63] The Lancet Respiratory Medicine (2018). Opening the black box of machine learning. Lancet Respir. Med..

[64] Luz C., Vollmer M., Decruyenaere J., Nijsten M., Glasner C., Sinha B. (2020). Machine learning in infection management using routine electronic health records: Tools, techniques, and reporting of future technologies. Clin. Microbiol. Infect..

[65] Maugeri A., Barchitta M., Agodi A. (2020). A Clustering Approach to Classify Italian Regions and Provinces Based on Prevalence and Trend of SARS-CoV-2 Cases. Int. J. Environ. Res. Public Health.

[66] Maugeri A., Barchitta M., Battiato S., Agodi A. (2020). Modeling the Novel Coronavirus (SARS-CoV-2) Outbreak in Sicily, Italy. Int. J. Environ. Res. Public Health.

[67] Maugeri A., Barchitta M., Battiato S., Agodi A. (2020). Estimation of Unreported Novel Coronavirus (SARS-CoV-2) Infections from Reported Deaths: A Susceptible–Exposed–Infectious–Recovered–Dead Model. J. Clin. Med..

[68] Maugeri A., Barchitta M., Battiato S., Agodi A. (2020). Estimation of unreported SARS-CoV-2 cases in Italy using a Susceptible-Exposed-Infectious-Recovered-Dead model. J. Glob. Health.

[69] Peiffer-Smadja N., Rawson T., Ahmad R., Buchard A., Georgiou P., Lescure F.-X., Birgand G., Holmes A. (2020). Corrigendum to ‘machine learning for clinical decision support in infectious diseases: A narrative review of current applications’ clinical microbiology and infection (2020) 584–595. Clin. Microbiol. Infect..

[70] Ripoli A., Sozio E., Sbrana F., Bertolino G., Pallotto C., Cardinali G., Meini S., Pieralli F., Azzini A.M., Concia E. (2020). Personalized machine learning approach to predict candidemia in medical wards. Infection.

[71] Amer A.Y.A., Vranken J., Wouters F., Mesotten D., Vandervoort P., Storms V., Luca S., Vanrumste B., Aerts J.-M. (2019). Feature Engineering for ICU Mortality Prediction Based on Hourly to Bi-Hourly Measurements. Appl. Sci..

