# Supplementary material for: Early Prediction of Seven-Day Mortality in Intensive Care Unit Using a Machine Learning Model: Results from the SPIN-UTI Project
Source: J Clin Med. 2021 Mar 2;10(5):992. doi: 10.3390/jcm10050992 (PMC7957866; doi:10.3390/jcm10050992)
Supplement: Supplementary file 1 [file jcm-10-00992-s001.pdf]

Supplementary Files

Early prediction of seven-day mortality in Intensive Care Unit using a machine learning model: results from the SPIN-UTI project

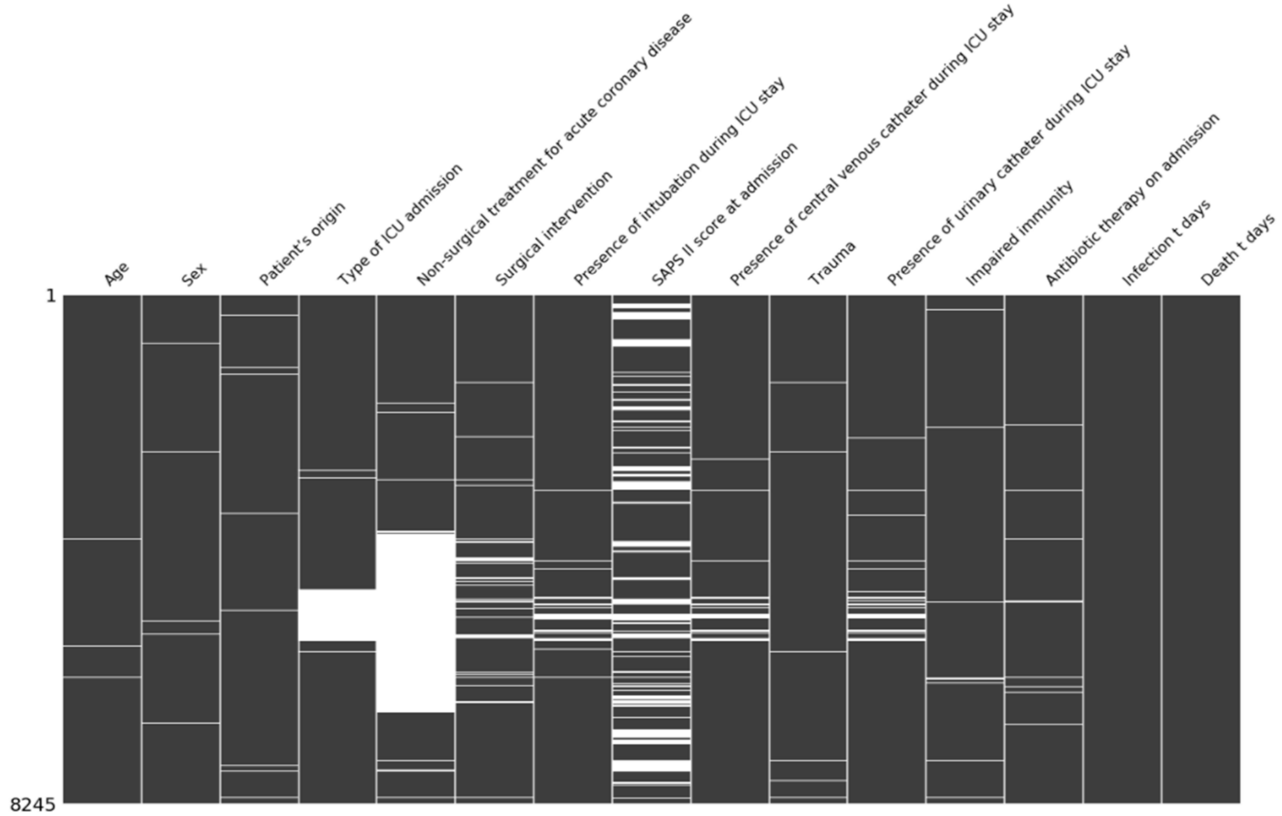

Figure S1. Matrix of missing values

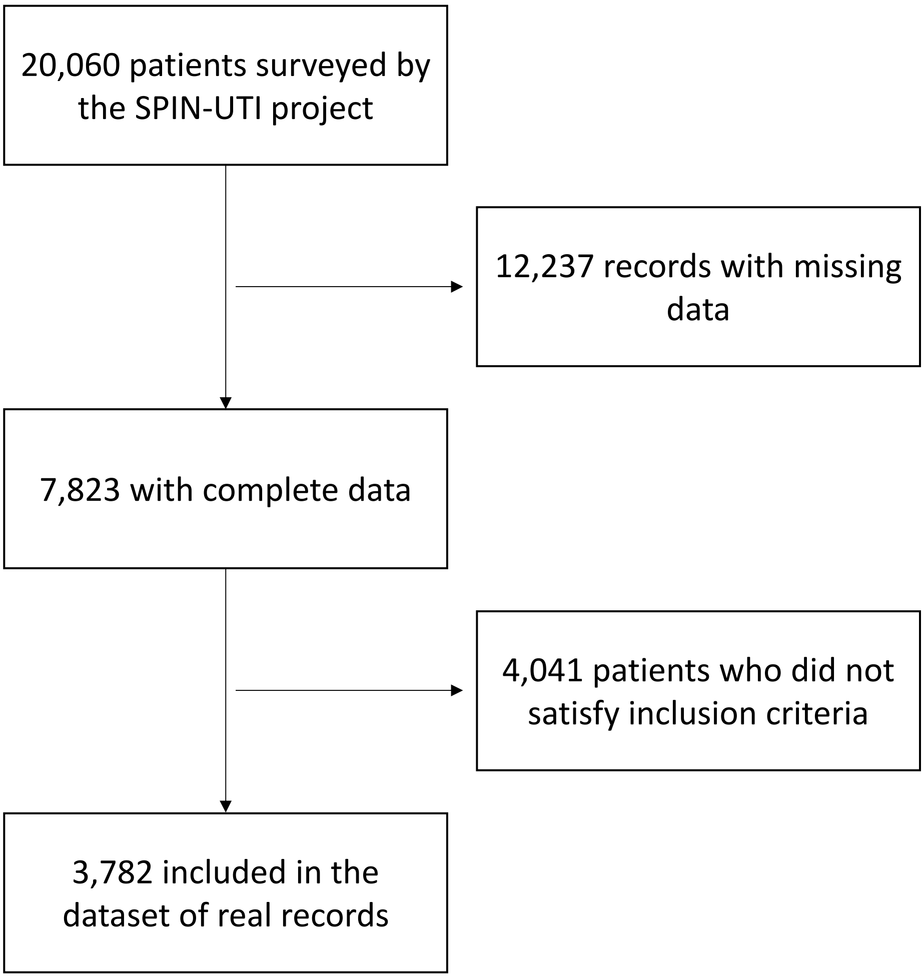

Figure S2. Selection of records with complete data satisfying inclusion criteria.

A

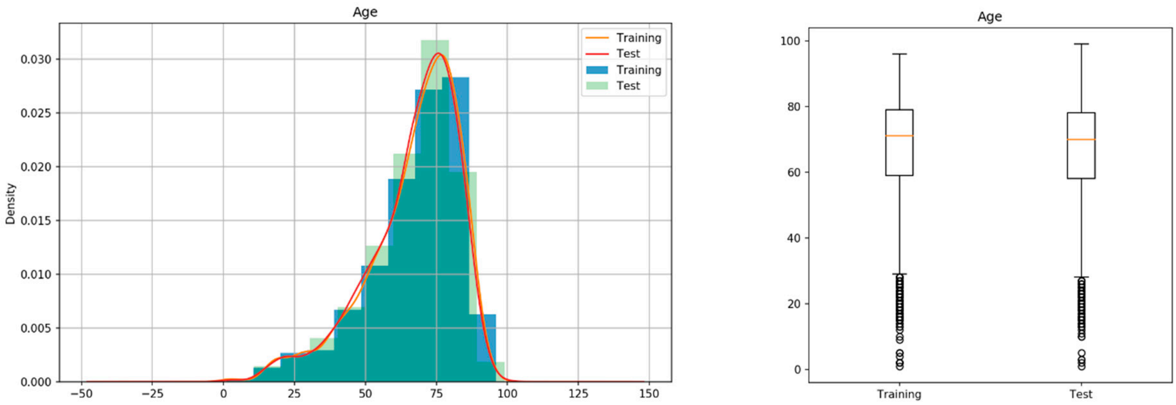

B

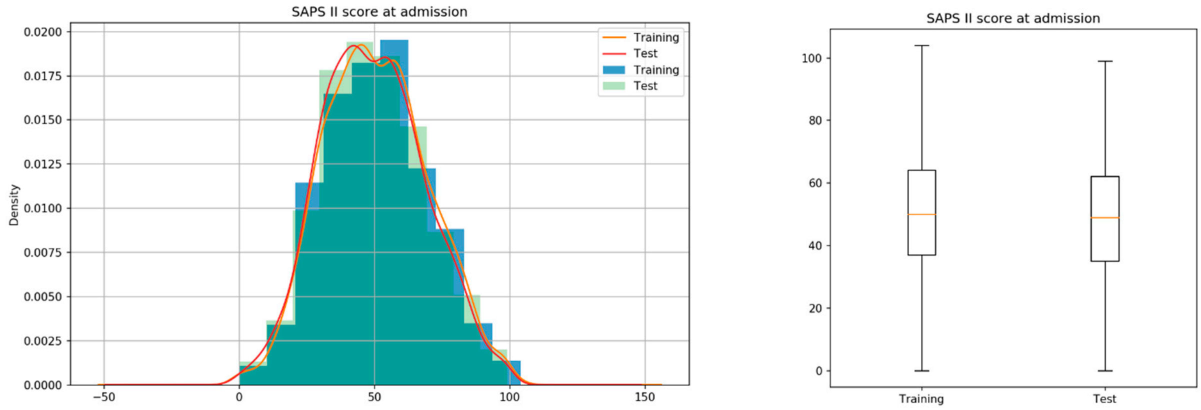

Figure S3. Comparison of Age (A) and SAPS II score (B) distributions between Training and Test sets

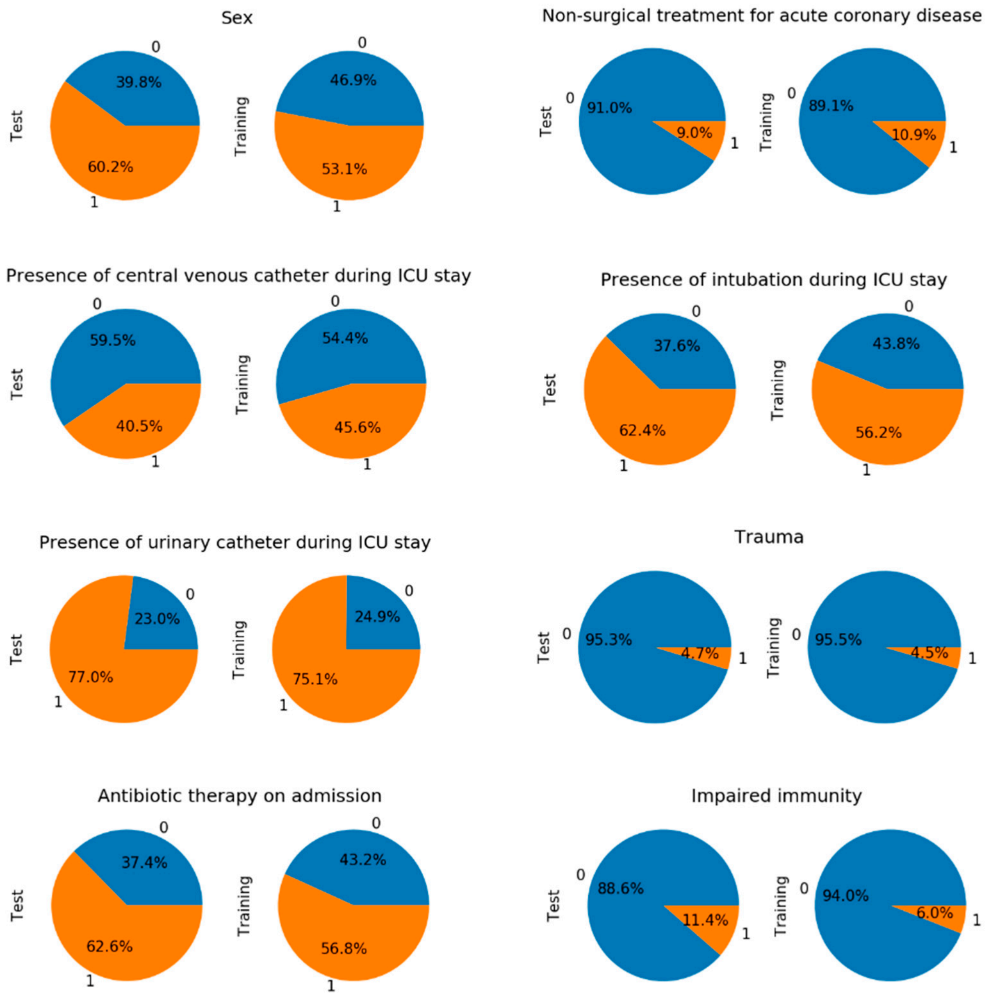

Figure S4. Comparison of dichotomous variables between Training and Test sets.

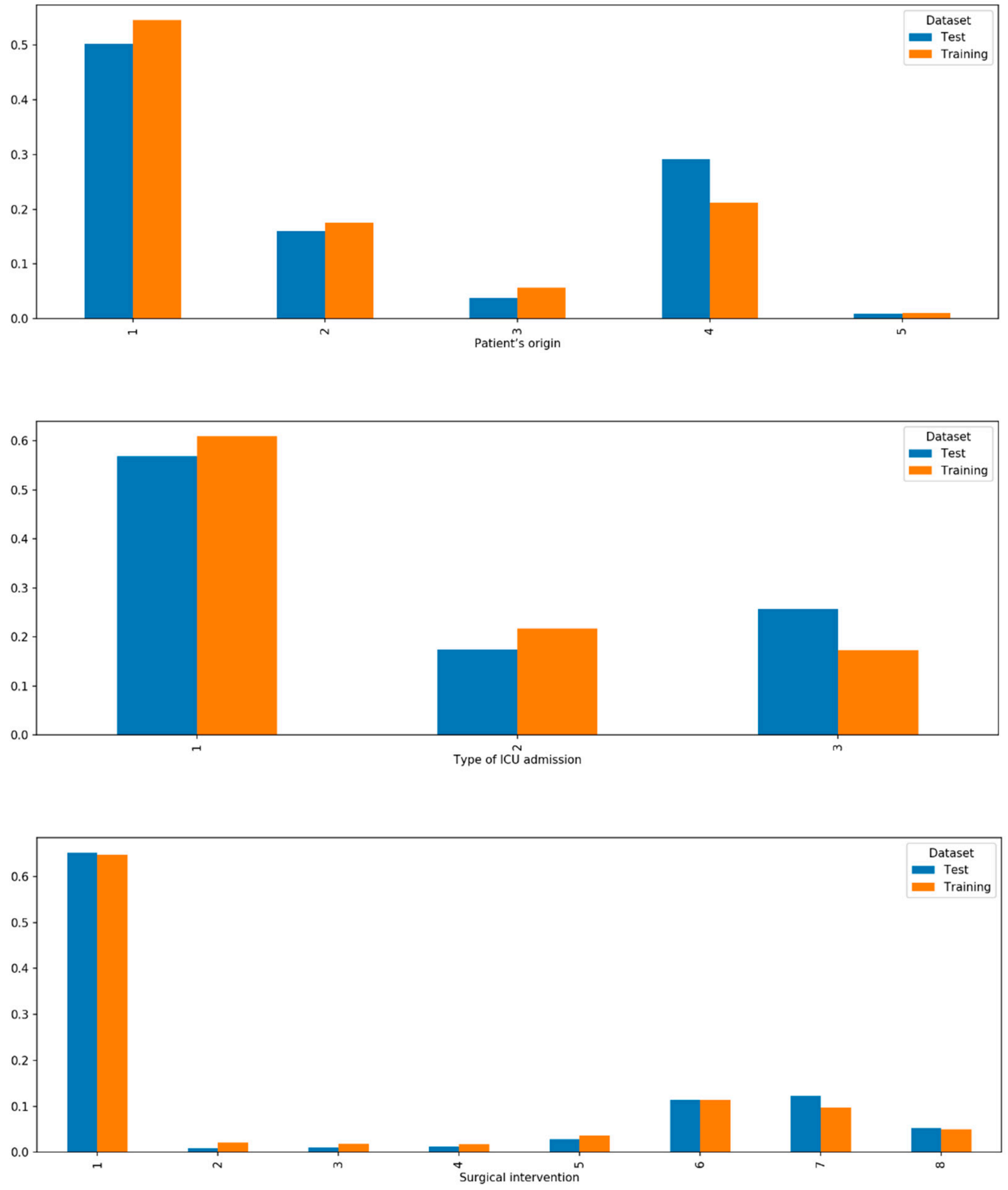

Figure S5. Comparison of categorical variables between training and test datasets.

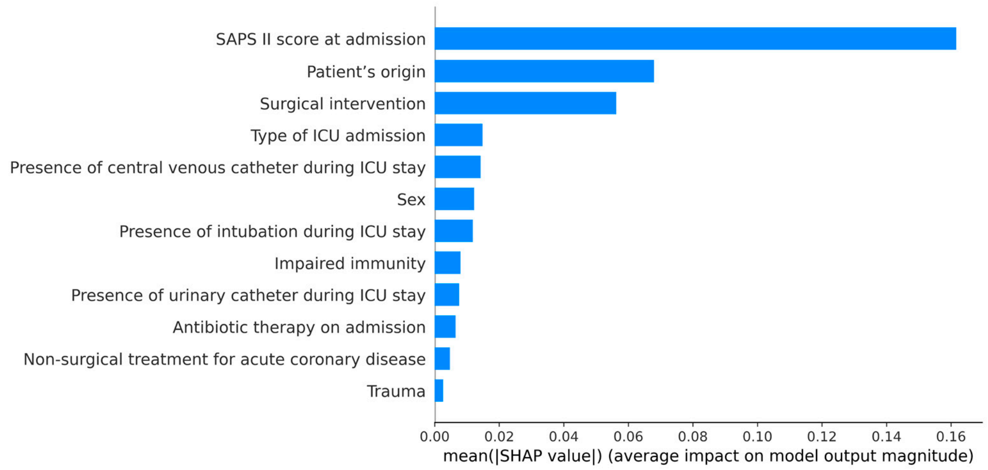

Figure S6. Shapley plot showing the contribution of each predictor to the SVM model output.

Table S1. Composition of training and test sets

Outcome	Training Set	Test set
**Class 0 (Alive patients)**	2,596 with imputation of missing data	2,907 real records
**Class 1 (Dead patients)**	1193 total<br>(662 with imputation of missing data and 1,131 after class balancing)	875 real records
**Total**	4,589 synthetic records	3,782 real records

Table S2. Coordinates of the ROC curve of logistic regression model with SAPS II alone

SAPS II values	Sensitivity	1-Specificity	SAPS II values	Sensitivity	1-Specificity	SAPS II values	Sensitivity	1-Specificity
**1**	0.997	0.998	34	0.899	0.751	67	0.333	0.148
**2**	0.995	0.998	35	0.895	0.729	68	0.318	0.135
**3**	0.995	0.997	36	0.879	0.708	69	0.303	0.125
**4**	0.994	0.997	37	0.869	0.688	70	0.293	0.115
**5**	0.994	0.996	38	0.857	0.672	71	0.272	0.108
**6**	0.993	0.994	39	0.848	0.649	72	0.258	0.099
**7**	0.993	0.992	40	0.839	0.628	73	0.247	0.092
**8**	0.992	0.990	41	0.827	0.604	74	0.224	0.085
**9**	0.992	0.987	42	0.814	0.582	75	0.211	0.079
**10**	0.992	0.986	43	0.806	0.561	76	0.197	0.073
**11**	0.990	0.984	44	0.779	0.539	77	0.178	0.065
**12**	0.990	0.982	45	0.765	0.521	78	0.161	0.057
**13**	0.987	0.980	46	0.750	0.501	79	0.152	0.052
**14**	0.985	0.973	47	0.731	0.479	80	0.144	0.048
**15**	0.983	0.971	48	0.718	0.461	81	0.131	0.042
**16**	0.981	0.967	49	0.704	0.440	82	0.121	0.035
**17**	0.976	0.961	50	0.693	0.422	83	0.104	0.030

**18**	0.975	0.958	51	0.678	0.404	84	0.095	0.025
**19**	0.971	0.952	52	0.667	0.387	85	0.087	0.024
**20**	0.969	0.946	53	0.649	0.369	86	0.072	0.021
**21**	0.967	0.941	54	0.634	0.347	87	0.064	0.017
**22**	0.965	0.933	55	0.619	0.329	88	0.055	0.015
**23**	0.962	0.924	56	0.583	0.306	89	0.050	0.014
**24**	0.958	0.915	57	0.563	0.289	90	0.048	0.011
**25**	0.955	0.899	58	0.541	0.272	91	0.039	0.010
**26**	0.953	0.890	59	0.512	0.258	92	0.035	0.009
**27**	0.950	0.875	60	0.486	0.245	93	0.031	0.008
**28**	0.947	0.859	61	0.471	0.231	94	0.025	0.007
**29**	0.937	0.845	62	0.446	0.212	95	0.025	0.006
**30**	0.934	0.829	63	0.413	0.200	96	0.023	0.004
**31**	0.927	0.810	64	0.394	0.187	97	0.018	0.003
**32**	0.919	0.791	65	0.377	0.172	98	0.016	0.003
**33**	0.909	0.772	66	0.353	0.159	99	0.011	0.002

Supplementary Methods

Data imputation

For replacing missing values, different imputation methods (i.e. the replacement of missing values with 0, mean, median or mode values and regression imputation) are commonly used. To do that, we used a *K-Nearest Neighbor* (K-NN) imputation method to recover part of the missing values for continue and categorical variables, according to Malarvizhi and Thanamani [1]. The K-NN method is based on the assumption that a point value can be approximated by the values of the points that are closest to it, based on the other variables [2]. It is useful for dealing with all kind of missing values whose distribution is unknown. In our study, we applied the algorithm for every different target variable considering Euclidean distance in the feature space for non-binary variables and Jaccard distance for dichotomic variables. In particular, the Jaccard distance is complementary to the Jaccard coefficient, defined as the size of the intersection divided by the size of the union of the sample sets.

$$J(A,B) = |A \cap B| \,/\, |A \cup B|$$
$$0 \le J(A,B) \le 1$$
$$d_j(A,B) = 1 - J(A,B)$$

Applying two cycles of 1-NN imputation separately to the two classes of data, death patients or not, we recovered 3258 records, approximately the 73% of the incomplete ones. After imputation, all available data were included in the analysis.

Support Vector Machine model

Datasets are often not linearly separable even in a feature space, not allowing to satisfy all the constraints in the minimization problem of SVM [3]. To solve this issue, *Slack variables* are introduced to allow certain constraints to be violated. By choosing very large slack variable values we could find a degenerate solution which would lead to the model overfitting. To penalize the assignment of too large slack variables, the penalty is introduced in the classification objective:

$$C\sum_{i=1}^{N} \varepsilon_i$$

- $\boldsymbol{\varepsilon_i}$**,** indicates "slack variables", one for each datapoint *i*, to allow certain constraints to be violated;
- $\boldsymbol{C}$, indicates a tuning parameter that controls the trade-off between the penalty of slack variables ε_i and the optimization of the margin. High values of C penalize slack variables leading to an hard margin, whereas low values of C lead to a soft margin, that is a bigger corridor which allows certain training points inside at the expense of misclassifying some of them. In particular, C parameter sets the confidence interval range of the learning model.

The RBF kernel function expression on two sample, x and x', is defined as $\mathrm{K}(\mathrm{x},\mathrm{x}') = \exp\left(-\gamma||\mathrm{x}-\mathrm{x}'||^2\right)$ where $||\mathrm{x}-\mathrm{x}'||^2$ is the squared Euclidean distance between the two feature vectors and $\boldsymbol{\gamma}$ is a free parameter. The RBF can be

applied to a dataset through the choice of two parameters, C and γ. The classifier performance of SVM depends on the choice of these two parameters. A Grid Search method was used to find the optimal parameters of the RBF for SVM. This method considered m values in C and n values in γ, according to the M×N combination of C and γ [4], by training different SVM using a K-fold cross validation. Here, to optimize the f1-score of the positive class, we used a Grid Search on a 5-fold cross validation. The analyses were performed using Python and Support Vector Classification (SVC) from Sklearn 0.22.1.

References

1. Malarvizhi, R.; Thanamani, A. K-nearest neighbor in missing data imputation. Int J Eng Res Dev 5(1):05–07 2012.
2. Obadia, Y. The use of KNN for missing values. https://towardsdatascience.com/the-use-of-knn-for-missing-values-cf33d935c637, 2017.
3. Cortes, C.; Vapnik, V. Support-Vector Networks. Mach. Learn. 20, 3 (September 1995), 273–297.
4. Han, S.; Qubo, C.; Meng, H. Parameter selection in SVM with RBF kernel function. World Automation Congress 2012, Puerto Vallarta, Mexico, 2012, pp. 1-4, 2012.
